# Efficacy of traditional Chinese Medicine-based biological extracts in the treatment of oral lichen planus

**DOI:** 10.3389/fcimb.2026.1746366

**Published:** 2026-07-15

**Authors:** Xiangyu Chen, Lekai Wang, Chaojun Xu, Caiyan Gu, Caohui He, Lu Sun, Xinhai Yu

**Affiliations:** 1 Hunan Provincial Key Laboratory of Regional Hereditary Birth Defects Prevention and Control, Changsha Hospital for Maternal & Child Health Care Affiliated to Hunan Normal University, Changsha, Hunan, China; 2 Department of Stomatology, YiYang Central Hospital, YiYang, Hunan, China

**Keywords:** biological extracts, immunology, oral lichen planus, pharmacology, traditional Chinese medicine

## Abstract

**Objective:**

Our network meta-analysis aimed to evaluate the efficacy of traditional Chinese Medicine(TCM)-based biological extracts treatment for patients with oral lichen planus (OLP).

**Methods:**

A systematic search of PubMed, Embase and Web of Science was conducted to identify randomized controlled trials evaluating the efficacy of TCM-based biological extracts in treating OLP. A network meta-analysis was then performed on the eligible trials, and the TCM-based biological extracts were ranked based on their effectiveness in OLP treatment using the Surface Under the Cumulative Ranking (SUCRA) scores.

**Results:**

In total, 22 articles were included in the meta-analysis. Direct comparisons showed that TCM-based biological extracts treatment significantly reduced visual analog scale (VAS) scores in OLP patients with an *MD* of -0.86 (95% CI: -1.36 to -0.37, *P* < 0.01). Network meta-analysis shows chamomile, curcumin, echinacea, and honey were effective in reducing clinical scores. Meanwhile, chamomile, echinacea, and honey were significant in reducing VAS scores.

**Conclusions:**

This study provides strong evidence for the efficacy of TCM-based biological extracts in the treatment of OLP through network meta-analysis, and in particular, TCM-based biological extracts such as echinacea and honey demonstrated significant advantages in relieving pain and improving clinical symptoms.

## 1 Introduction

Oral lichen planus represents a chronic autoimmune disorder primarily affecting the epithelial, basal, and underlying lamina propria layers of the oral mucosa, characterized by its persistent nature (Omal et al., 2012). The precise etiology of lichen planus remains elusive, with the prevailing hypothesis implicating the upregulation of cytokines, notably leukocytokines, interleukin-1, tumor necrosis factor, interferon, and matrix metalloproteinase-9, as key contributors to its pathogenesis (Lavanya et al., 2011). Recent researches stressed OLP as an immune-mediated disorder, orchestrated by T-cells in response to antigenic stimuli, highlighting its complex immune-driven etiology (El-Howati et al., 2023). Also, researchers found that hepatitis C virus and human papillomavirus infections are risk factors for OLP (Lodi et al., 2004; Lodi et al., 2010). A recent systematic review and meta-analysis indicated a global prevalence of approximately 1% for OLP, accompanied by a discernible upward trend in incidence rates (González-Moles et al., 2021). The incidence of OLP was slightly higher in women than in men (Cassol-Spanemberg et al., 2018), and the peak age of onset tends to be between 30 and 60 years (Le Cleach and Chosidow, 2012). OLP was predominantly of atrophic and erosive type, which occurred in the buccal mucosa, and tend to be recurrent. It can cause a great impact on the quality of life of patients, and even become cancerous (Radwan-Oczko et al., 2018).

Currently, there is no standardized treatment for OLP (Louisy et al., 2024). Current management strategies for OLP prioritize the mitigation of inflammation and alleviation of accompanying symptomatology, particularly pain and burning sensations. Given the immune-mediated etiology of OLP, anti-inflammatory, immunomodulator, and immunosuppressant therapies have been used to address the underlying disease mechanisms (Yuan et al., 2022). The use of topical glucocorticosteroids is considered to be the first-line drug for the treatment of OLP (Gupta et al., 2017). Furthermore, clinical trials have explored a range of pharmacological interventions, including calmodulin neural phosphatase inhibitors, retinoids, photodynamic therapy, curcumin, vitamin D, and hyaluronic acid (Andabak-Rogulj et al., 2023). However, long-term use of western drugs such as hormones and immunosuppressants can cause a large number of adverse reactions, including decreased immunity, atrophy of oral mucosa, and easy recurrence. TCM-based biological extracts, based on the concept of wholeness and the differentiation of syndromes, can better avoid the above problems by regulating the function of the organs to treat diseases (Zheng et al., 2011).

TCM-based biological extracts not only have anti-inflammatory, immunomodulatory, antioxidant and other biological activities, but also play a unique role in the treatment of OLP by regulating immune response and inhibiting cytokine secretion (Dai et al., 2007). For example, licorice extract has been shown to have significant anti-inflammatory effects and can alleviate the clinical symptoms of OLP by inhibiting the release of interleukin-1 and tumor necrosis factor and reducing the local inflammatory response (Fu et al., 2013). In addition, curcumin, as a natural antioxidant, has been proven to have a significant effect on reducing oral mucosal damage and promoting wound healing (Liu et al., 2024). Extracts such as resveratrol and green tea polyphenols have also shown potential to control OLP inflammation and promoting immune balance through their antioxidant and anti-inflammatory properties (D'Amico et al., 2023).

The advantages of TCM-based biological extracts are their naturalness, low toxicity and few side effects, which make them a promising aid in modern medical treatment (Katiyar et al., 2012). More importantly, compared with traditional Western drug treatment, TCM-based biological extracts can usually maintain stable efficacy over a longer period of time, reducing drug dependence and immunosuppressive side effects. These advantages have led to the gradual inclusion of TCM-based biological extracts as an adjunct treatment option for OLP, especially in patients who cannot tolerate conventional Western medicine or who wish to seek long-term results.

Several RCTs using TCM-based biological extracts for the treatment of OLP have been conducted, and their efficacy has been superior to that of western drug treatments. However, to date, there are still few RCT-based meta-analyses of TCM-based biological extracts for OLP (Radwan-Oczko et al., 2018; You et al., 2020), which limits our comprehensive understanding of their therapeutic effects. Network meta-analysis, as an advanced statistical method, is capable of comparing the relative effectiveness of multiple treatment options simultaneously, thus providing clinicians with a more precise basis for decision-making (Rücker and Schwarzer, 2017).

Therefore, this study aimed to critically evaluate the existing RCTs of TCM-based biological extracts for OLP through network meta-analysis, to further clarify their efficacy and safety, and to explore the potential value of TCM-based biological extracts as an adjunctive therapy in the management of OLP.

## 2 Methods

This systematic review adhered strictly to a predefined protocol, adhering to the Preferred Reporting Items for Systematic Reviews and Meta-Analyses (PRISMA) guidelines, along with the PRISMA extension for Network Meta-Analyses (NMA) in healthcare interventions, thereby ensuring academic rigor and succinctness in the reporting process.

### 2.1 Data sources and search strategy

A comprehensive search of PubMed, EMBASE and Web of Science databases up to August 1, 2025 was conducted to retrieve all pertinent studies, utilizing multiple synonyms and abbreviations as keywords. To ensure comprehensiveness, we meticulously examined the reference lists of previous systematic reviews. This process was followed by independent screening of titles/abstracts and full texts by two reviewers, with ineligible studies being excluded at each consecutive stage. Discrepancies were resolved through consensus.

### 2.2 Selection criteria

The inclusion criteria for studies encompassed RCTs featuring participants clinically or histologically confirmed with OLP without comorbid oral diseases. Interventions solely involving TCM-based biological extracts were considered. For studies involving both OLP and/or oral lichenoid lesions, exclusive OLP data was extracted; otherwise, the study was excluded. Additionally, comparisons solely between different modalities of the same corticosteroid were also omitted from analysis.

### 2.3 Outcomes

The primary efficacy outcomes comprised (Omal et al., 2012): the clinical score, quantified as the mean variation utilizing the Thongprasom^’^s scale (Thongprasom et al., 1992), ranging from 0 (absence of lesions) to 5 (severe erosive areas > 1 cm^2^ accompanied by white striae), and (Lavanya et al., 2011) the pain score, measured as the mean change on a visual analogue scale, a 10-cm horizontal line calibrated from 0 (no pain) to 10 (maximum pain intensity).

### 2.4 Data extraction and quality assessment

Data and pertinent details from eligible studies were extracted independently by two reviewers, and the corresponding authors were contacted for clarification or to obtain missing information, ensuring a rigorous and comprehensive data collection process. The RCTs’ data were systematically categorized into study demographics, population characteristics, intervention specifics, outcome definitions, and measures. The risk of bias (ROB) within each study was independently evaluated by two reviewers utilizing RevMan version 5.4.1.

### 2.5 Data synthesis and statistical analysis

Utilizing the “meta” package in R, traditional pairwise meta-analyses were conducted to derive pooled estimates for direct comparisons. Heterogeneity was quantified via Cochran’s Q statistic and I^2^ index, with a Q test *P* value < 0.05 or *I*^2^ > 50% suggestive of significant heterogeneity, necessitating the adoption of a random-effects model.

The “netmeta” package in R facilitated NMA, integrating direct and indirect intervention comparisons into a comprehensive network-based comparison. Network model inconsistency was evaluated using heterogeneity statistics across treatment designs, with a Q test *P* value < 0.05 or *I^2^* > 50% indicative of inconsistency. A node-splitting approach further assessed inconsistency between direct and indirect estimates, where *P* < 0.05 suggested a discrepancy, potentially necessitating the application of a random-effects model.

Network plots were constructed to visualize intervention comparisons, with NMA outcomes presented in forest plots and league tables. Publication bias was evaluated through comparison-adjusted funnel plot analysis, and its presence was confirmed by Egger’s test (*P* < 0.05). Continuous outcomes, specifically clinical and pain scores, were quantified as mean difference (MD) with 95% confidence intervals (CIs). All statistical analyses were conducted using R (version 4.4).

The Surface Under the Cumulative Ranking (SUCRA) curves were utilized to determine the intervention hierarchy within the network meta-analysis, with higher SUCRA scores indicative of superior clinical effectiveness for OLP treatment. A sensitivity analysis was conducted, excluding studies with a high risk of bias to further validate the findings.

## 3 Results

### 3.1 Characteristics and quality of studies

**Figure 1:**
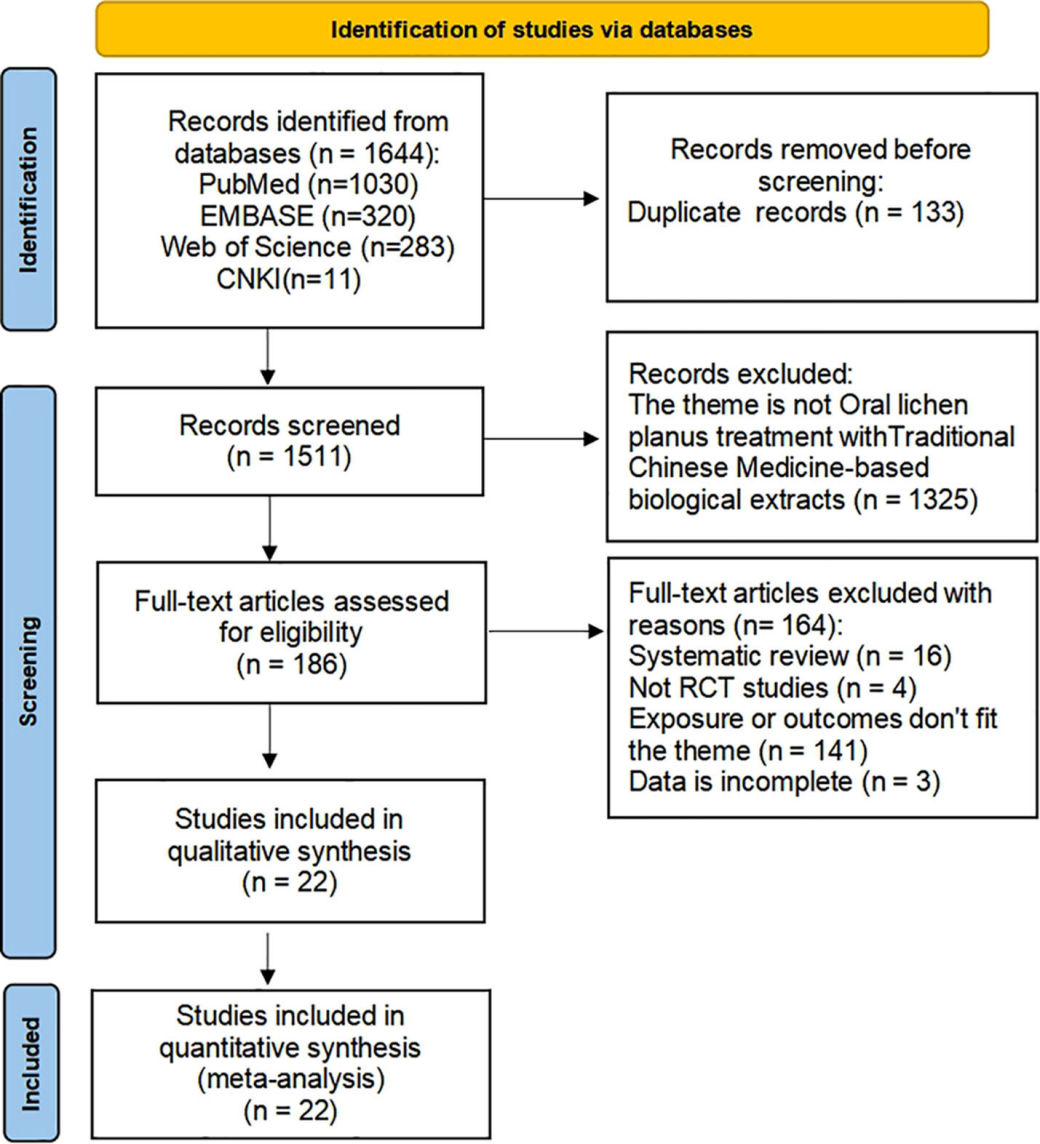
PRISMA flow chart.

Following the elimination of 133 duplicates, we conducted a title and abstract screening of 1,500 articles. Subsequently, 186 full-text articles were evaluated for eligibility, leading to the exclusion of 164 studies and ultimately yielding 22 RCTs (Choonhakarn et al., 2008; Salazar-Sánchez et al., 2010; Mansourian et al., 2011; Saawarn et al., 2011; Chainani-Wu et al., 2012; Amirchaghmaghi et al., 2015; Keshari and Karthikeya Patil, 2015; Kia et al., 2015; Amirchaghmaghi et al., 2016; Lopez and Aznar-Cayuela, 2016; Pratibha et al., 2016; Kushwaha et al., 2017; Keller and Kragelund, 2018; Kia et al., 2020; Eita et al., 2021; Fathy Samhan and Mohamed Abdelhalim, 2021; Hazzaa et al., 2021; Bhatt et al., 2022; Chaitanya et al., 2022; Ghobadi et al., 2022; Saberi et al., 2022; Vaidya et al., 2023) for NMA (The PRISMA flowchart **Figure 1**).

The characteristics of the 22 included RCTs (n = 1029) were described in **Table 1**. All included studies were double-blind RCTs. The 22 included RCTs containing 9 different TCM-based biological extracts interventions. Of these, five studies in which aloe vera (AV) was used in the experimental group; six studies used curcumin in the experimental group; five studies used lycopene in the experimental group; the remaining six interventions were Echinacea, Spirulina, Honey, Probiotic, Chamaemelum, Quercetin. The studies represented 8 different countries (**Table 1**). Seven studies were conducted in Iran, 6 studies in India, 3 studies in Egypt, 2 studies in Spain, the remaining 4 studies were implemented in the United States, Denmark, Nepal and Thailand respectively. All of the included studies were two-arm studies. All participants were confirmed to have symptomatic or erosive OLP based on clinical and/or histopathological diagnostic criteria. The treatment duration was 2–16 weeks.

**Table 1:** Specific information on included articles.

Author (year)	Country	Study design	Diagnostic criteria	Sample size after exclusion	Study comparison	Duration of treatment	Outcome: clinical score	Outcome: pain score
Vaidya (Vaidya et al., 2023)	India	Single-center double-blind RCT	Clinical and histopathological diagnosis	52	Aloe vera vs Clobetasol Propionate	8 weeks	NA	mean: 2.33 ; 1.70 sd: 2.17 ; 1.55
Ghobadi (Ghobadi et al., 2022)	Iran	Single-center double-blind RCT	Clinical and histopathological diagnosis	28	Curcumin vs PBO	4 weeks	mean: 11.79 ; 15.97 sd: 6.79 ; 7.44	mean: 0.36 ; 1.79 sd: 0.49 ; 1.44
Saberi (Saberi et al., 2022)	Iran	Single-center double-blind RCT	Clinical and histopathological diagnosis	70	Echinacea vs PBO	5 weeks	mean: 1.20 ; 2.60 sd: 1.25 ; 0.55	mean: 1.77 ; 4.74 sd: 1.97 ; 1.19
Chaitanya (Chaitanya et al., 2022)	India	Single-center double-blind RCT	Clinical and histopathological diagnosis	60	Spirulina vs Steroid	8 weeks	NA	mean: 2.63 ; 3.90 sd: 3.09 ; 2.91
Bhatt (Bhatt et al., 2022)	India	Single-center double-blind RCT	Clinical and histopathological diagnosis	60	Aloe vera vs low-level laser therapy	8 weeks	mean: 0.33 ; 0.60 sd: 0.12 ; 0.12	mean: 1.63 ; 3.03 sd: 0.20 ; 0.22
Hazzaa (Hazzaa et al., 2021)	Egypt	Single-center double-blind RCT	Clinical and histopathological diagnosis	40	Lycopene vs Prednisolone	8 weeks	NA	mean: 2.35 ; 2.60 sd: 0.98 ; 1.69
Eita (Eita et al., 2021)	Egypt	Single-center double-blind RCT	Clinical and histopathological diagnosis	20	Lycopene vs Prednisolone	8 weeks	mean: 3.30 ; 1.90 sd: 1.83 ; 0.88	NA
Samhan (Fathy Samhan and Mohamed Abdelhalim, 2021)	Egypt	Single-center double-blind RCT	Clinical and histopathological diagnosis	46	Honey vs PBO	4 weeks	mean: 1.15 ; 2.78 sd: 0.38 ; 0.45	mean: 2.17 ; 4.73 sd: 1.31 ; 1.38
Kia (Kia et al., 2020)	Iran	Single-center double-blind RCT	Clinical and histopathological diagnosis	57	Curcumin vs Prednisolone	4 weeks	mean: 2.34 ; 1.83 sd: 1.14 ; 0.92	mean: 2.69 ; 2.33 sd: 2.89 ; 2.03
Keller (Keller and Kragelund, 2018)	Denmark	Single-center double-blind RCT	Clinical and histopathological diagnosis	22	Probiotic vs PBO	16 weeks	mean: 8.10 ; 7.2 sd: 7.8 ; 5.7	mean: 19.6 ; 34.2 sd: 22.1 ; 21.3
Kushwaha (Kushwaha et al., 2017)	Nepal	Single-center double-blind RCT	Clinical and histopathological diagnosis	28	Lycopene vs Prednisolone	8 weeks	mean: 2.15 ; 0.73 sd: 1.68 ; 1.58	mean: 0.23 ; 0.076 sd: 0.44 ; 0.26
Shekhawat 2016	India	Single-center double-blind RCT	Clinical and histopathological diagnosis	50	Lycopene vs Levamisole	8 weeks	mean: 1.44 ; 2.24 sd: 0.65 ; 1.05	mean: 1.88 ; 3.96 sd: 1.56 ; 1.71
Maryam 2016	Iran	Single-center double-blind RCT	Clinical and histopathological diagnosis	20	Curcumin vs PBO	4 weeks	mean: 1.08 ; 1.5 sd: 0.66 ; 1.06	mean: 0.33 ; 0.13 sd: 0.65 ; 0.35
Jornet 2016	Spain	Single-center double-blind RCT	Clinical and histopathological diagnosis	55	Chamaemelum vs PBO	4 weeks	mean: 1.09 ; 1.64 sd: 0.50 ; 0.70	mean: 1.88 ; 4.31 sd: 1.30 ; 1.90
Maryam 2015	Iran	Single-center double-blind RCT	Clinical and histopathological diagnosis	30	Quercetin vs PBO	4 weeks	mean: 3.23 ; 1.33 sd: 3.47 ; 1.87	mean: 0.23 ; 0.46 sd: 0.43 ; 0.83
Seid 2015	Iran	Single-center double-blind RCT	Clinical and histopathological diagnosis	50	Curcumin vs Prednisolone	4 weeks	mean: 2.64 ; 2.95 sd: 1.29 ; 0.97	mean: 2.64 ; 1.76 sd: 2.98 ; 1.78
Keshari (Keshari and Karthikeya Patil, 2015)	India	Single-center double-blind RCT	Clinical and histopathological diagnosis	27	Curcumin vs Prednisolone	2 weeks	mean: 0.40 ; 0.41 sd: 0.63 ; 0.66	mean: 1.13 ; 2.58 sd: 1.64 ; 2.66
Chainani-Wu (Chainani-Wu et al., 2012)	USA	Single-center double-blind RCT	Clinical and histopathological diagnosis	20	Curcumin vs PBO	2 weeks	mean: 1.98 ; 2.42 sd: 3.01 ; 3.01	mean: 3.93 ; 5.18 sd: 1.72 ; 1.29
Arash Mansourian (Mansourian et al., 2011)	Iran	Single-center double-blind RCT	Clinical and histopathological diagnosis	46	AV vs Prednisolone	4 weeks	NA	mean: 0.83 ; 0.87 sd: 0.11 ; 0.10
Saawarn (Saawarn et al., 2011)	India	Single-center double-blind RCT	Clinical and histopathological diagnosis	30	Lycopene vs PBO	8 weeks	NA	mean: 7.60 ; 16.30 sd: 9.20 ; 18.30
N. Salazar-Sanchez (Salazar-Sánchez et al., 2010)	Spain	Single-center double-blind RCT	Clinical and histopathological diagnosis	64	AV vs PBO	6 weeks	NA	mean: 2.70 ; 3.40 sd: 2.80 ; 3.00
C. Choonhakarn (Choonhakarn et al., 2008)	Thailand	Single-center double-blind RCT	Clinical and histopathological diagnosis	54	AV vs PBO	8 weeks	mean: 4.18 ; 4.1 sd: 0.42 ; 0.47	mean: 6.78 ; 6.91 sd: 0.71 ; 0.67

We performed a network meta-analysis of two outcome (clinical score and pain score). Separately, 17 articles reported clinical score and 22 articles reported pain score. The detailed study characteristics pertaining to each outcome are summarized in **Table 1**. The risk of bias assessment for the included studies is presented in **Supplementary Figures 1** and **S2**.

### 3.2 Pairwise meta-analysis

#### 3.2.1 Clinical score

**Figure 2:**
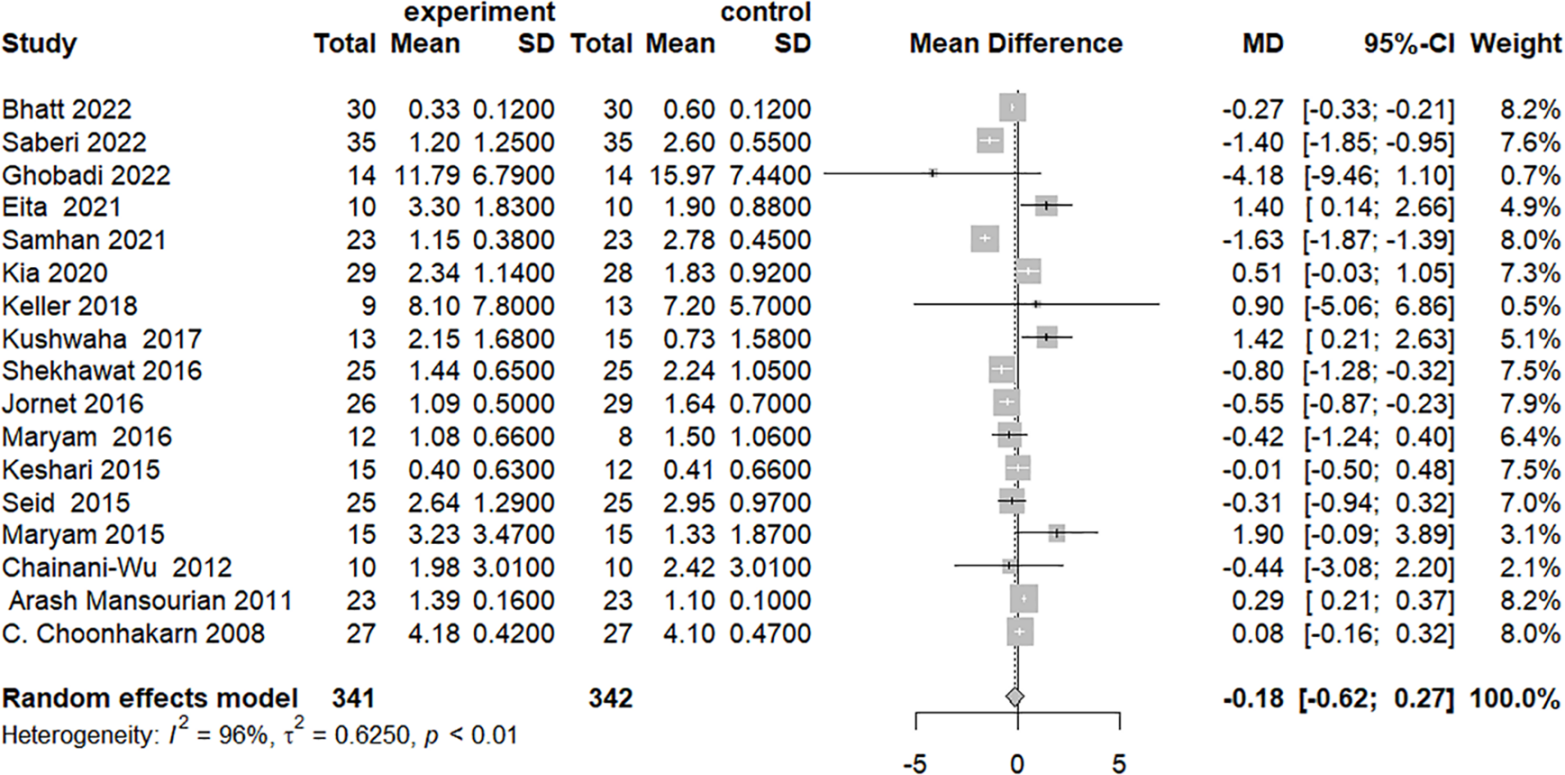
Forest plot of pairwise clinical score.

A total of 17 RCTs comparing 12 interventions were included in the analysis for the clinical score outcome. Meta-analytic pooling of 17 RCTs yielded a summary *MD* of -0.18 (95% *CI* -0.62–0.27, *P*>0.05)(see **Figure 2**).

Data from 3 RCTs (n = 160) which used AV were pooled for oral clinical score (**Supplementary Figure 3**). No significant difference was observed for AV versus Control [*MD*:0.03,95%*CI*:(-0.31,0.37)]. Data from 6 RCTs (n = 202) which used Curcumin were pooled for oral clinical score (**Supplementary Figure 4**). There was no significant difference between Curcumin and Control [*MD*:-0.04,95%*CI*:(-0.44,0.36)]. Data from 3 RCTs (n = 98) which used Lycopene were pooled for oral clinical score (**Supplementary Figure 5**). No significant difference was found between Lycopene and Control [*MD*:0.58,95%*CI*:(-0.95,2.11)].

#### 3.2.2 Pain score

**Figure 3:**
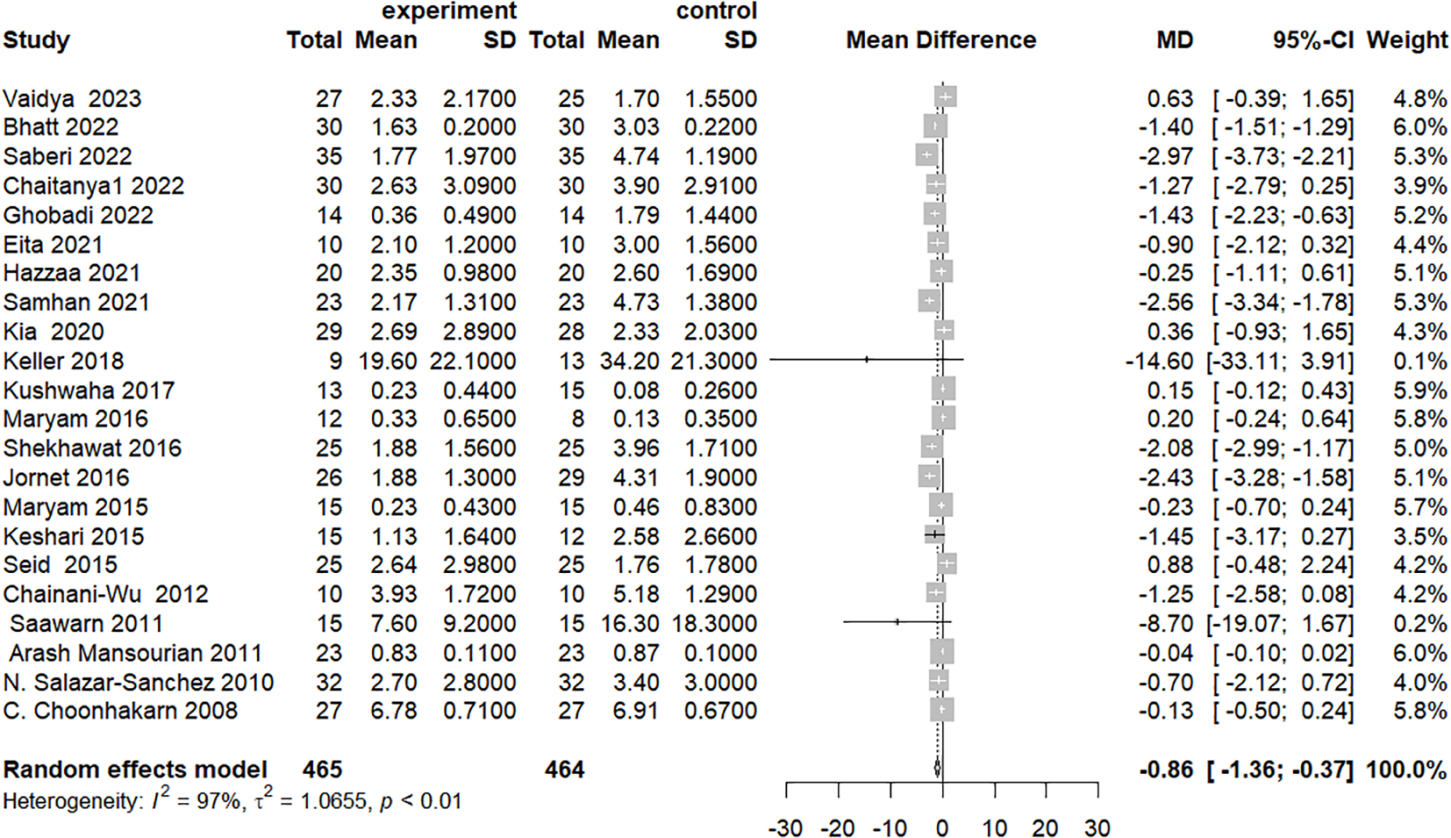
Forest plot of pairwise pain score.

A total of 22 RCTs comparing 15 interventions were incorporated for the pain score outcome. A meta-analysis encompassing these RCTs revealed a summary *MD* of -0.86 (95% *CI* -1.36–0.37, *P* < 0.01)(see **Figure 3**).

Data from 5 RCTs (n = 276) which used AV were pooled for pain score (**Supplementary Figure 6**). No significant difference was observed for AV versus Control [*MD*:-0.36,95%*CI*:(-1.06,0.33)]. Data from 6 RCTs (n = 202) which used Curcumin were pooled for pain score (**Supplementary Figure 7**). No significant difference was observed for AV versus Control [*MD*:-0.41,95%*CI*:(-1.23,0.40)]. Data from 5 RCTs (n = 168) which used Lycopene were pooled for pain score (**Supplementary Figure 8**). No significant difference was observed for AV versus Control [*MD*:-0.79,95%*CI*:(-1.79,0.22)].

### 3.3 Network meta-analysis

#### 3.3.1 Clinical score

**Figure 4:**
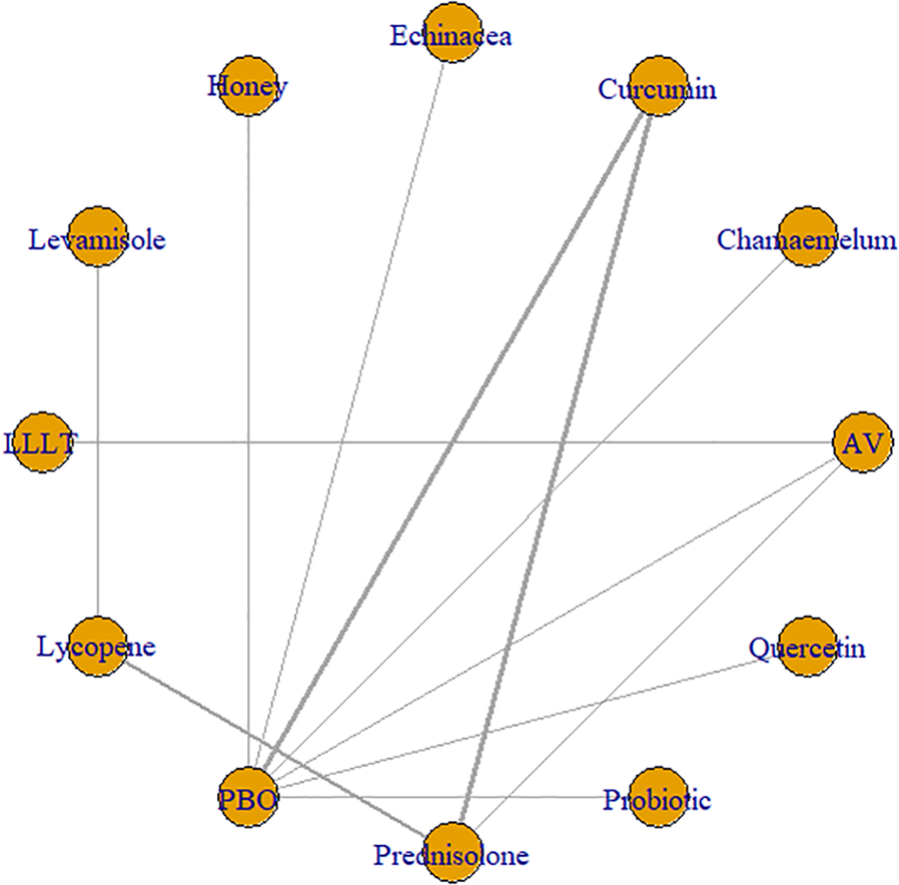
Network plot of clinical score.

A total of 17 RCTs evaluating 12 interventions were included for the analysis of the clinical score outcome. The network plot was provided in **Figure 4**.

Network meta-analysis suggested that AV[*MD*:1.64,95%*CI*:(0.18,2.92)], Chamaemelum [*MD*:-2.42,95%*CI*:(-4.31,-0.11)], Curcumin [*MD*:-2.20,95%*CI*:(-3.83,-0.56)], Echinacea [*MD*:-3.32,95%*CI*:(-5.74,-0.84)], Honey [*MD*:-3.56,95%*CI*:(-5.96,-1.07)], Levamisole [*MD*:2.23,95%*CI*:(0.71,3.74)] and Lycopene [*MD*:1.44,95%*CI*:(0.33,2.51)] showed statistically significant in clinical score for OLP (see **Supplementary Table 1**). When ranked, the top three were Levamisole [SUCRA 89.46], Quercetin [SUCRA 87.22], Lycopene [SUCRA 76.72].

In the subgroup analysis of clinical scores, only the comparison between PBO and other TCM-based biological extracts showed statistical significance, with Echinacea [MD:-1.40,95%CI:(-2.40,-0.40)], Honey [MD:-1.60,95%CI:(-2.60,-0.71)] (see **Supplementary Figure 9**).

#### 3.3.2 Pain score

**Figure 5:**
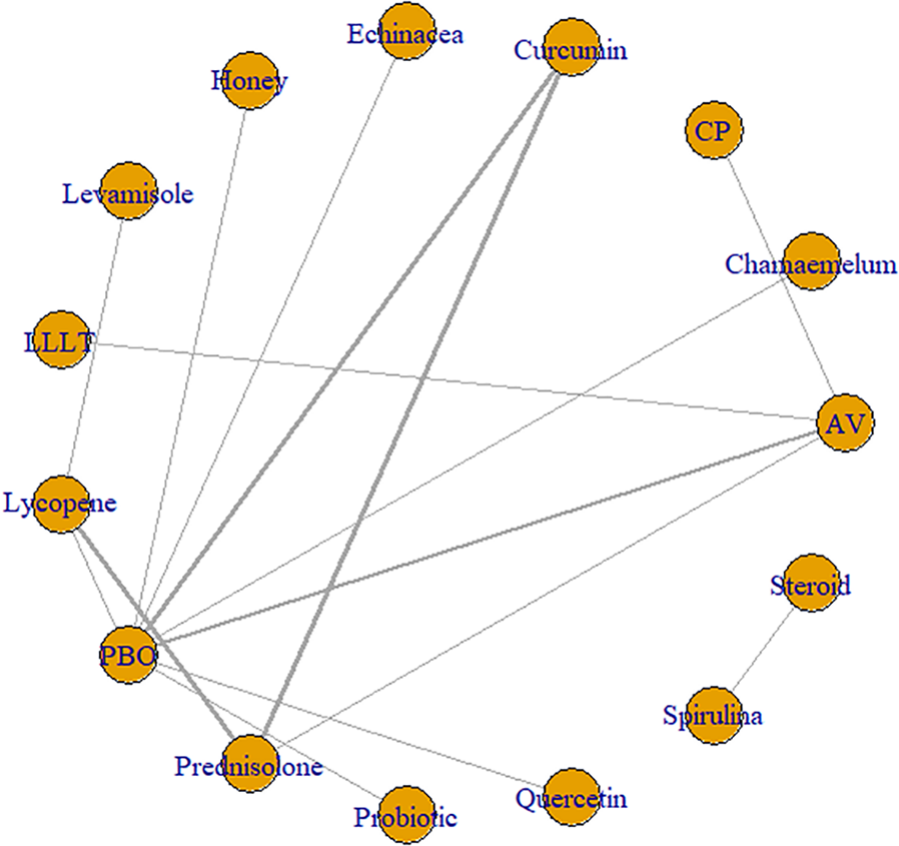
Network plot of pain score.

A total of 22 RCTs involving 15 interventions were considered for the pain score outcome. The network plot illustrating the comparisons was presented in **Figure 5**.

Network meta-analysis suggested that AV[*MD*:2.61,95%*CI*:(0.33,4.89)], Chamaemelum [*MD*:-2.43,95%*CI*:(-4.54,-0.28)], Control [*MD*:2.95,95%*CI*:(0.46,4.64)] and Curcumin [*MD*:2.55,95%*CI*:(0.26,4.83)] showed statistically significant in pain score for OLP(see **Supplementary Table 2**). When ranked, the top three were Control [SUCRA 88.22], Quercetin [SUCRA 75.20], AV [SUCRA 73.21].

In the subgroup analysis of pain scores, only the comparison between control and other TCM-based biological extracts showed statistical significance, with Chamaemelum [MD:-2.40,95%CI:(-4.50,-0.28)], Echinacea [MD:-3.00,95%CI:(-5.10,-0.88)], Honey [MD:-2.60,95%CI:(-4.60,-0.46)] (see **Supplementary Figure 10**).

### 3.4 Network consistency and sensitivity analysis

For clinical score, the test of global inconsistency showed no evidence of inconsistency within clinical score; and the network evidence for pain score has no closed-loop structure and therefore does not require a consistency test. Our results suggested that no publication bias both in clinical score (t = -0.52, *P* = 0.61)and pain score (t = -1.18, *P* = 0.25). Sensitivity analyses demonstrated that the exclusion of any individual study did not significantly alter the pooled effect sizes for both the clinical score and pain score outcomes in OLP (see **Supplementary Figures 11**, **S12**).

## 4 Discussion

This study provides insight into the efficacy of TCM-based biological extracts in the treatment of OLP, particularly in relieving patients’ pain (as measured by VAS scores) and improving clinical symptoms, by means of a Network Meta-analysis (NMA) based on RCTs. Our findings not only provide strong evidence support for the use of TCM-based biological extracts in the treatment of OLP, but also demonstrate the unique strengths and contributions of this study by comparing the results of other meta-analyses.

Direct comparisons showed that TCM-based biological extracts treatment significantly reduced VAS scores in OLP patients with an *MD* of -0.86 (95% *CI*: -1.36 to -0.37, *P* < 0.01), a finding that was consistent with previous findings on TCM-based biological extracts in the area of pain management (Zhu et al., 2024). The significant reduction in VAS scores was a direct reflection of the role of herbs in relieving OLP patients’ subjective pain perception and brings positive improvement in patients’ quality of life. Through network meta-analysis, we further revealed the specific effects and rankings of chamomile, curcumin, echinacea, and honey in reducing clinical and VAS scores. Honey demonstrated the best efficacy in both analyses, and its unique antibacterial, anti-inflammatory, and tissue-repair-promoting properties may have set the stage for its superior performance (Wijesinghe et al., 2009). Echinacea was a close second, with its rich polyphenolic compounds showing potent activity in anti-inflammatory and antioxidant properties, which may be one of the reasons for its remarkable efficacy (Burlou-Nagy et al., 2022). Chamomile and curcumin also performed well, and their respective sedative, anti-inflammatory, and immunomodulatory functions provide a multi-pathway intervention strategy for the treatment of OLP (Peng et al., 2021). The studies included in this research originate from different countries, and there are certain variations in treatment duration and sample sizes, which led to high heterogeneity in the forest plot. We performed a sensitivity analysis on this issue, and the results showed that removing studies one by one did not change the robustness of the findings (see **Supplementary Figures 11**, **S12**). This may reflect the complexity of “syndrome differentiation and treatment” in TCM-based biological extracts. The differences between the results of the pairwise meta-analysis and the network meta-analysis in this study may stem from the limited direct comparative evidence and small sample size in the pairwise analysis (e.g., only three studies investigated interventions using AV and lycopene, with the smallest sample size being 20 participants), whereas NMA leverages indirect evidence to produce more statistically robust results. Furthermore, by incorporating subgroup analysis, NMA can control for certain heterogeneity, thereby improving the reliability of the findings.

Compared to other meta-analyses focusing on OLP treatments (You et al., 2020; Yuan et al., 2022), this study demonstrates strengths in several ways. First, we employed a network meta-analysis approach that enabled us to simultaneously compare multiple treatment options and assess the relative efficacy among them, thus providing more comprehensive and in-depth insights. Second, our focus on TCM-based biological extracts fills a gap in the systematic evaluation of this field in OLP treatment, providing clinicians and patients with more diverse treatment options. In addition, the high quality of the RCTs included in this study and the relative rigor of the study design made the reliability of the results somewhat assured. Despite the important findings of this study, there are some limitations. First, the complexity and diversity of TCM-based biological extracts make it difficult to fully quantify the differences in efficacy between different formulas. Second, due to limited data, the safety indicators of herbal medicine for OLP were not discussed in this study, so the evaluation is limited. Furthermore, although the SUCRA value can reflect the relative ranking of efficacy, it does not directly indicate clinical significance or the magnitude of absolute effects, necessitating a comprehensive evaluation. SUCRA rankings are particularly sensitive to small sample sizes and heterogeneity. The variations in sample sizes (20–70) and differences in treatment duration (2 weeks-16weeks) among the studies included in this paper may be possible reasons for the higher SUCRA values in the control group.

Although this study mainly focused on the assessment of efficacy, we also briefly discussed the possible mechanisms of TCM-based biological extracts in the treatment of OLP. Traditional Chinese medicine believes that the pathogenesis of OLP is closely related to the accumulation of dampness and heat in the stomach, and stagnation of qi and blood stasis (Zhou, 2012). Therefore, clearing heat and removing toxins has become an important principle in the treatment of OLP with TCM-based biological extracts (Deng et al., 2022). These TCM-based biological extracts, chamomile and curcumin, alleviate the inflammatory symptoms of OLP by inhibiting the release of inflammatory mediators and attenuating the immune response (Amirchaghmaghi et al., 2016; Saberi et al., 2022). Homeopathic medicines are highly diluted versions of various substances, including botanical extracts. Mucosal (oral and genital) lichen planus has been found to respond favorably to homeopathic therapies in case series (Mousavi et al., 2009; Nwabudike et al., 2019).In addition, patients with OLP often suffer from localized blood circulation disorders, resulting in insufficient blood supply to the diseased area, further aggravating the condition (Zhu et al., 2024). Honey and echinacea can improve local blood circulation and promote inflammation absorption and lesion repair (Fathy Samhan and Mohamed Abdelhalim, 2021; Saberi et al., 2022). Meanwhile, OLP is also an immune disease, and its pathogenesis is related to the abnormal activation of the immune system. Various components in TCM-based biological extracts have immunomodulatory effects, which can regulate the T-cell-mediated immune response and attenuate the immune reaction. For example, *Paeonia lactiflora* total glucoside, an active ingredient extracted from the dried rhizome of Paeonia lactiflora, has immunomodulatory, antioxidant, anti-inflammatory and analgesic effects, and improves blood circulation (Fu et al., 2013).

In conclusion, the mechanism of TCM-based biological extracts for the treatment of OLP may involve several aspects, including anti-inflammatory, antioxidant, immunomodulatory, and promotion of tissue repair. These mechanisms are intertwined and work together in the pathologic process of OLP to achieve therapeutic effects.

This study provides strong evidence for the efficacy of TCM-based biological extracts in the treatment of OLP through network meta-analysis, and in particular, TCM-based biological extracts such as echinacea and honey demonstrated significant advantages in relieving pain and improving clinical symptoms. In the future, more high-quality, large-sample RCTs should be conducted to further validate these findings and to explore the integration of TCM-based biological extracts with modern medical treatments to provide more comprehensive and effective treatment options for OLP patients.

## Data Availability

The original contributions presented in the study are included in the article/**Supplementary Material**. Further inquiries can be directed to the corresponding author.

## Supplementary material

Supplementary Figure 1 Graph risk of bias for clinical score studies.

Supplementary Figure 2 Graph risk of bias for pain score studies.

Supplementary Figure 3 Forest plot of clinical scores for aloe vera versus control group.

Supplementary Figure 4 Forest plot of clinical scores for curcumin versus control group.

Supplementary Figure 5 Forest plot of clinical scores for lycopene versus control group.

Supplementary Figure 6 Forest plot of pain scores for aloe vera versus control group.

Supplementary Figure 7 Forest plot of pain scores for curcumin versus control group.

Supplementary Figure 8 Forest plot of pain scores for lycopene versus control group.

Supplementary Figure 9 Subgroup analysis of clinical scores.

Supplementary Figure 10 Subgroup analysis of pain scores.

Supplementary Figure 11 Sensitivity analysis of clinical scores.

Supplementary Figure 12 Sensitivity analysis of pain scores.

Supplementary Table 1 The league table of clinical score.

Supplementary Table 2 The league table of pain score.
