# Supplementary material for: Efficacy of traditional Chinese Medicine-based biological extracts in the treatment of oral lichen planus
Source: Front Cell Infect Microbiol. 2026 Jul 15;16:1746366. doi: 10.3389/fcimb.2026.1746366 (PMC13414812; doi:10.3389/fcimb.2026.1746366)
Supplement: Supplementary Figure 1 — Graph risk of bias for clinical score studies. [file DataSheet1.docx]

Figure S1 Graph risk of bias for clinical score studies


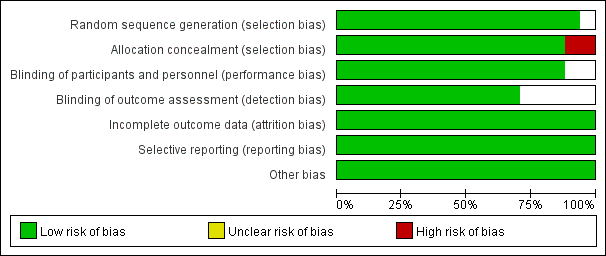


Figure S2 Graph risk of bias for pain score studies


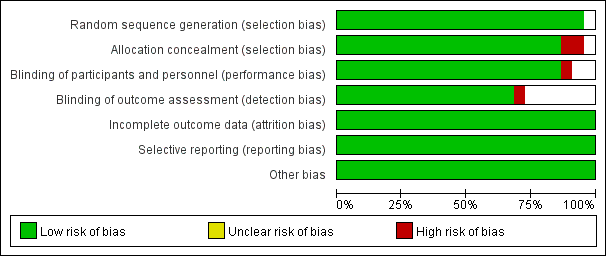


Figure S3 Forest plot of clinical scores for aloe vera versus control group


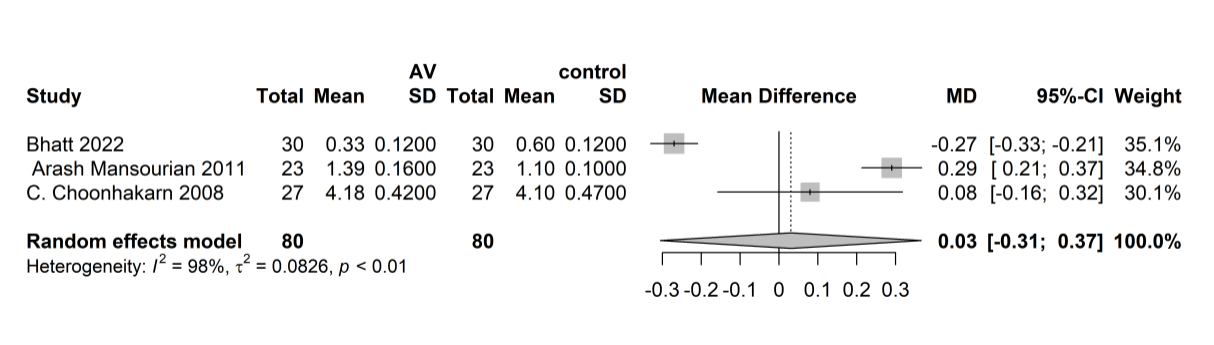


Figure S4 Forest plot of clinical scores for curcumin versus control group

Figure S5 Forest plot of clinical scores for lycopene versus control group
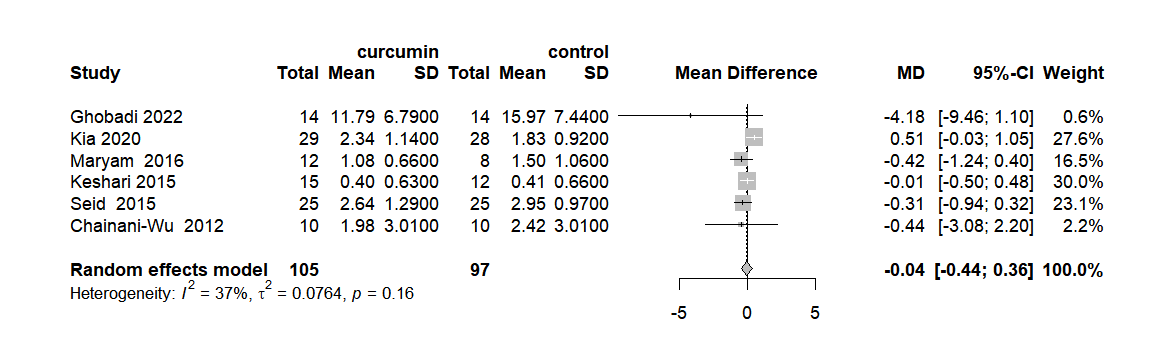


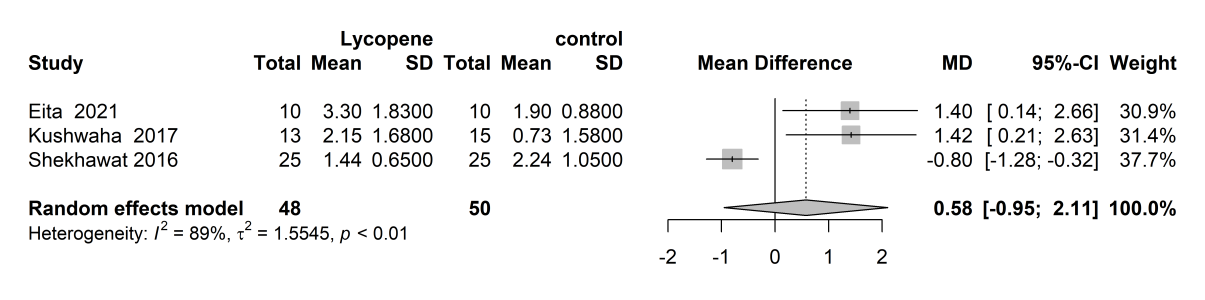
Figure S6 Forest plot of pain scores for aloe vera versus control group


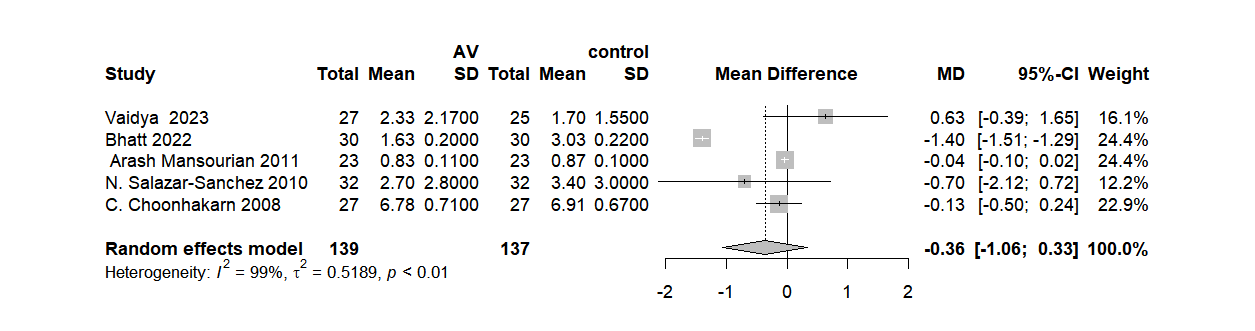
Figure S7 Forest plot of pain scores for curcumin versus control group


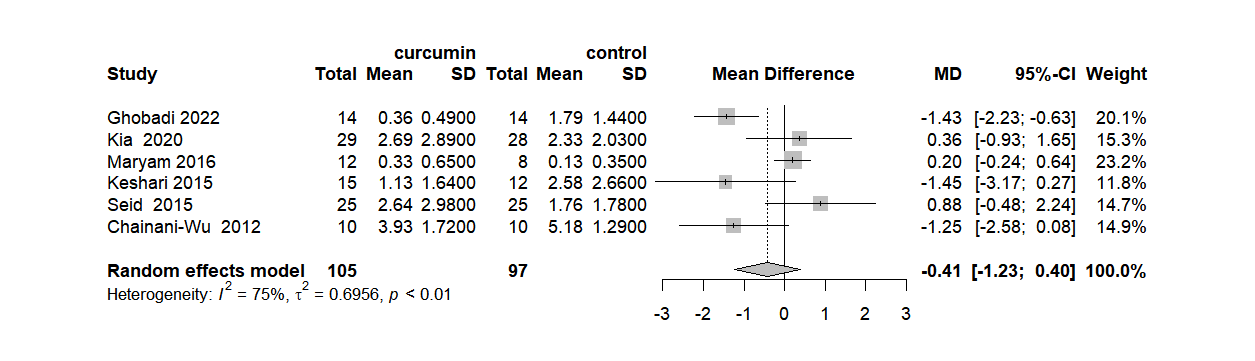


Figure S8 Forest plot of pain scores for lycopene versus control group


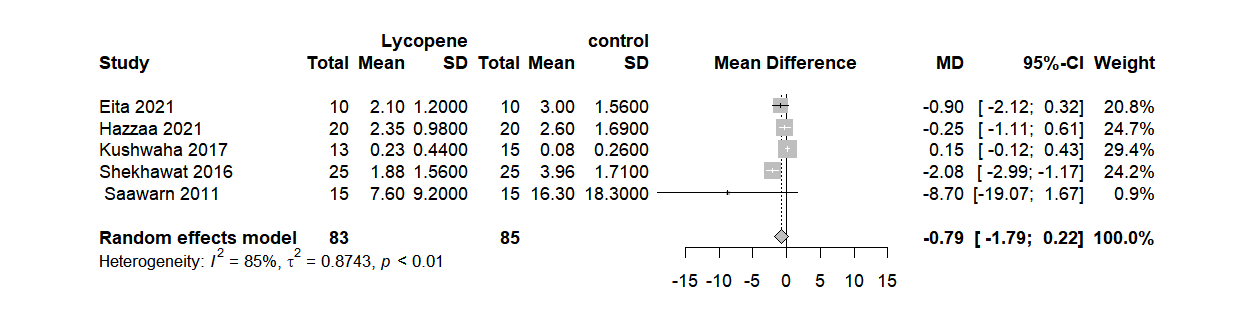
Figure S9 Subgroup analysis of clinical scores


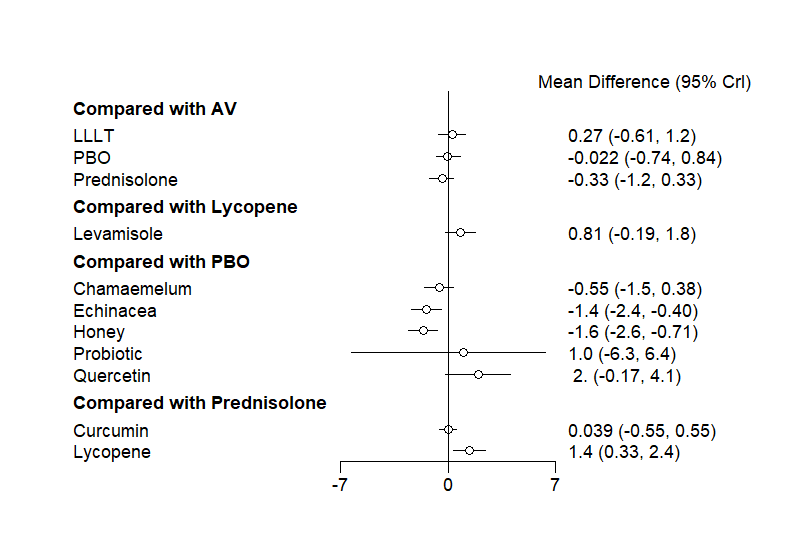


Figure S10 Subgroup analysis of pain scores


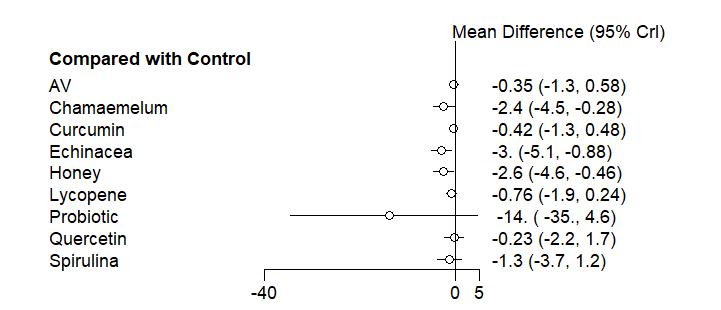


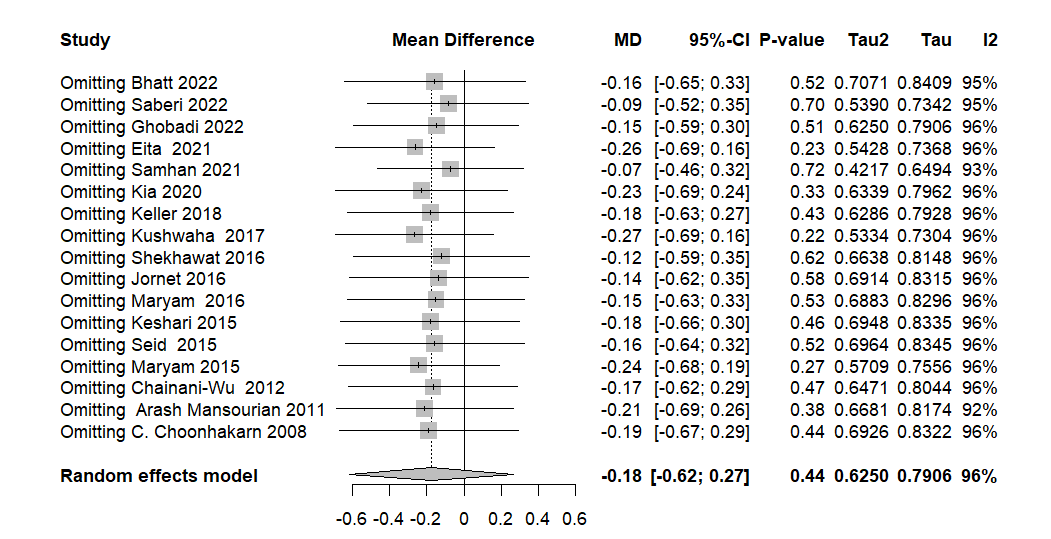
Figure S11 Sensitivity analysis of clinical scores

Figure S12 Sensitivity analysis of pain scores


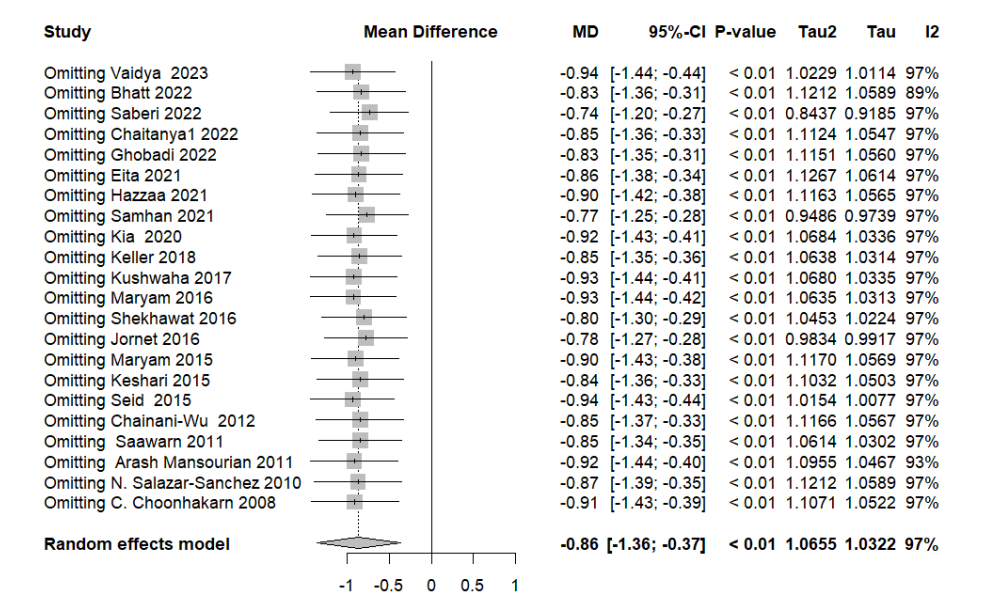


| AV |  |  |  |  |  |  |  |  |  |  |  |
| --- | --- | --- | --- | --- | --- | --- | --- | --- | --- | --- | --- |
| 0.56 (-0.93, 1.84) | Chamaemelum |  |  |  |  |  |  |  |  |  |  |
| 0.30 (-0.48, 1.44) | -0.25 (-1.40, 1.41) | Curcumin |  |  |  |  |  |  |  |  |  |
| 1.39 (-0.04, 2.70) | 0.83 (-0.65, 2.37) | 1.08 (-0.52, 2.28) | Echinacea |  |  |  |  |  |  |  |  |
| 1.64 (0.18, 2.92) | 1.08 (-0.42, 2.56) | 1.34 (-0.28, 2.48) | 0.25 (-1.27, 1.74) | Honey |  |  |  |  |  |  |  |
| -1.87 (-3.45, 0.01) | -2.42 (-4.31, -0.11) | -2.20 (-3.83, -0.56) | -3.26 (-5.18, -1.04) | -3.51 (-5.34, -1.20) | Levamisole |  |  |  |  |  |  |
| -0.27 (-1.30, 0.76) | -0.83 (-2.48, 0.96) | -0.57 (-2.14, 0.66) | -1.66 (-3.30, 0.11) | -1.92 (-3.47, -0.12) | 1.60 (-0.53, 3.47) | LLLT |  |  |  |  |  |
| -0.27 (-1.30, 0.76) | -1.63 (-3.22, 0.40) | -1.40 (-2.64, -0.15) | -2.46 (-4.04, -0.50) | -2.71 (-4.27, -0.73) | 0.80 (-0.31, 1.92) | -0.81 (-2.36, 1.01) | Lycopene |  |  |  |  |
| 0.01 (-0.97, 0.80) | -0.56 (-1.61, 0.53) | -0.30 (-1.45, 0.39) | -1.38 (-2.48, -0.33) | -1.63 (-2.66, -0.56) | 1.87 (-0.11, 3.46) | 0.28 (-1.16, 1.54) | 1.08 (-0.55, 2.30) | PBO |  |  |  |
| 0.34 (-0.40, 1.36) | -0.21 (-1.43, 1.46) | 0.04 (-0.61, 0.63) | -1.04 (-2.28, 0.57) | -1.30 (-2.49, 0.35) | 2.23 (0.71, 3.74) | 0.60 (-0.60, 2.08) | 1.44 (0.33, 2.51) | 0.34 (-0.42, 1.53) | Prednisolone |  |  |
| -1.24 (-6.80, 4.14) | -1.75 (-7.32, 3.63) | -1.58 (-7.20, 3.79) | -2.59 (-8.20, 2.81) | -2.82 (-8.41, 2.56) | 0.65 (-5.15, 6.30) | -0.96 (-6.61, 4.50) | -0.15 (-5.85, 5.34) | -1.20 (-6.66, 4.10) | -1.61 (-7.14, 3.76) | Probiotic |  |
| -1.24 (-6.80, 4.14) | -2.48 (-4.89, 0.04) | -2.29 (-4.69, 0.04) | -3.32 (-5.74, -0.84) | -3.56 (-5.96, -1.07) | -0.11 (-3.02, 2.71) | -1.69 (-4.14, 0.87) | -0.91 (-3.58, 1.70) | -1.93 (-4.17, 0.33) | -2.32 (-4.70, 0.01) | -0.74 (-6.77, 5.16) | Quercetin |

Table S1 The league table of clinical score

Table S2 The league table of pain score

| AV |  |  |  |  |  |  |  |  |  |
| --- | --- | --- | --- | --- | --- | --- | --- | --- | --- |
| 2.07 (-0.24, 4.39) | Chamaemelum |  |  |  |  |  |  |  |  |
| -0.35 (-1.26, 0.58) | -2.43 (-4.54, -0.28) | Control |  |  |  |  |  |  |  |
| 0.07 (-1.23, 1.40) | -2.02 (-4.27, 0.34) | 0.42 (-0.48, 1.32) | Curcumin |  |  |  |  |  |  |
| 2.61 (0.33, 4.89) | 0.53 (-2.37, 3.55) | 2.95 (0.88, 5.07) | 2.55 (0.26, 4.83) | Echinacea |  |  |  |  |  |
| 2.20 (-0.09, 4.48) | 0.13 (-2.83, 3.12) | 2.56 (0.46, 4.64) | 2.15 (-0.15, 4.41) | -0.41 (-3.37, 2.54) | Honey |  |  |  |  |
| 0.41 (-0.92, 1.87) | -1.67 (-3.96, 0.80) | 0.76 (-0.24, 1.85) | 0.34 (-0.99, 1.80) | -2.20 (-4.50, 0.20) | -1.79 (-4.06, 0.57) | Lycopene |  |  |  |
| 13.49 (-4.89, 34.39) | 11.40 (-7.02, 32.32) | 13.85 (-4.56, 34.63) | 13.41 (-5.12, 34.31) | 10.91 (-7.56, 31.73) | 11.30 (-7.29, 32.32) | 13.09 (-5.33, 33.91) | Probiotic |  |  |
| -0.11 (-2.27, 2.087) | -1.67 (-3.96, 0.80) | 0.23 (-1.73, 2.24) | -0.18 (-2.33, 2.03) | -2.71 (-5.64, 0.18) | -2.31 (-5.20, 0.57) | -0.53 (-2.79, 1.68) | -13.59 (-34.42, 4.83) | Quercetin |  |
| 0.93 (-1.73, 3.53) | -1.17 (-4.40, 2.11) | 1.27 (-1.20, 3.69) | 0.86 (-1.81, 3.44) | -1.69 (-4.88, 1.53) | -1.30 (-4.47, 1.88) | 0.50 (-2.22, 3.10) | -12.56 (-33.50, 5.74) | 1.03 (-2.12, 4.13) | Spirulina |
